# Accurate dating of stalagmites from low seasonal contrast tropical Pacific climate using Sr 2D maps, fabrics and annual hydrological cycles

**DOI:** 10.1038/s41598-021-81941-x

**Published:** 2021-01-26

**Authors:** Mohammadali Faraji, Andrea Borsato, Silvia Frisia, John C. Hellstrom, Andrew Lorrey, Adam Hartland, Alan Greig, David P. Mattey

**Affiliations:** 1 School of Environmental and Life Sciences, The University of Newcastle, Newcastle, NSW 2308 Australia; 2 School of Earth Sciences, The University of Melbourne, Melbourne, VIC 3010 Australia; 3 National Institute of Water and Atmospheric Research Ltd., Auckland, 1149 New Zealand; 4 Environmental Research Institute, School of Science, Faculty of Science and Engineering, University of Waikato, Hamilton, 3240 New Zealand; 5 Department of Earth Sciences, Royal Holloway University of London, Egham, TW20 0EX Surrey UK

**Keywords:** Palaeoclimate, Geochemistry

## Abstract

Tropical Pacific stalagmites are commonly affected by dating uncertainties because of their low U concentration and/or elevated initial ^230^Th content. This poses problems in establishing reliable trends and periodicities for droughts and pluvial episodes in a region vulnerable to climate change. Here we constrain the chronology of a Cook Islands stalagmite using synchrotron µXRF two-dimensional mapping of Sr concentrations coupled with growth laminae optical imaging constrained by in situ monitoring. Unidimensional LA-ICP-MS-generated Mg, Sr, Ba and Na variability series were anchored to the 2D Sr and optical maps. The annual hydrological significance of Mg, Sr, Ba and Na was tested by principal component analysis, which revealed that Mg and Na are related to dry-season, wind-transported marine aerosols, similar to the host-rock derived Sr and Ba signatures. Trace element annual banding was then used to generate a calendar-year master chronology with a dating uncertainty maximum of ± 15 years over 336 years. Our approach demonstrates that accurate chronologies and coupled hydroclimate proxies can be obtained from speleothems formed in tropical settings where low seasonality and problematic U–Th dating would discourage the use of high-resolution climate proxies datasets.

## Introduction

The South Pacific islands are highly vulnerable to the effects of both climate variability driven by El Niño–Southern Oscillation (ENSO)^1^ activity and climate change. However, understanding links between rainfall trends, rain intensity, episodic drought, and deluge against a backdrop of contemporary warming remains incomplete due to limited long, quantitative records^2^. Reducing uncertainties about the magnitude, frequency and duration of past droughts and pluvials is sufficient justification for obtaining well-dated palaeoproxy information from South Pacific speleothems. Speleothems (e.g. stalagmites, stalactites and flowstones) can provide long climate records outside the polar regions because they can be precisely dated and are relatively unaffected by post-depositional (diagenetic) modifications^3,4^. They also contain physical and chemical multi-proxy data that may reflect surface processes and impacts of climate variability and change. To date, speleothem material from South Pacific islands is notorious for having problems with reliable radiometric dating. This is a critical geochronology issue to surmount because accurately dated South Pacific speleothems could let us establish statistical relationships between drivers of regional rainfall variability, like ENSO, and local impacts on the environment^5,6^ and at the same time better define a phenomenon that affects billions of people worldwide.

Accurate U–Th dating using ion-counting multi-collector inductively coupled plasma-mass spectrometry (ICP-MS) is by far the most common method to establish the age of speleothem layers (cf. Hellstrom^7^ and references therein). Tropical island stalagmites, however, can be affected by large dating uncertainties related to their elevated and variable initial [^230^Th/^232^Th] ratio, a commonly reported but as yet poorly understood issue^8–10^. Accurate U–Th dating requires either insignificant or known Th^230^ activity at the time of speleothem crystal layers formation^7^, but incorporation of ^230^Th during deposition is common. An initial ^230^Th contamination is accompanied by a much larger amount of ^232^Th, and an assumed ^230^Th/^232^Th ratio is used in these cases to estimate initial ^230^Th activity.

The limitations of speleothem U–Th dating in this case can be overcome via acquiring precise relative age models by using high-frequency variations in visible growth laminae properties^11,12^, or in the cyclicity of their geochemical properties, such as trace element concentration^13–20^, or C and O isotope ratios^21,22^. The main assumptions when using physical laminae to optimize U–Th dating is that they are annual and that there are no growth interruptions. Similarly, refinement of U–Th dating using trace elements requires seasonal cyclicity, continuous speleothem growth and lateral homogeneity of trace element concentration. Chronologies based on stable C and O isotope ratios are limited by instrumental resolution and sample size.

Dating stalagmites using trace element cycles has recently been exploited by Nagra et al.^13^ to build chronology for annually laminated stalagmites with large U–Th ages uncertainties. Their study implied that trace element cycles work best to test the accuracy of annual lamina counting when seasonality in temperature and/or rainfall is transmitted to the stalagmite through variations in drip water chemistry. Seasonal chemical cycles have also confirmed the annual nature of stalagmite fluorescent laminae from monsoon Asia and indicate trace elements are a mechanism to improve dating in speleothems lacking visual or fluorescent lamination^20^. These studies used unidimensional laser ablation ICP-MS (LA-ICP-MS) tracts along the stalagmite vertical growth direction and assumed that the horizontal (annual) growth layers capture changes in parent-water chemistry in a laterally continuous and homogeneous manner. Only in this case, peaks and troughs identifying high-frequency trace element cycles along speleothem vertical growth axis transects (the time dimension) should show the same pattern and number. Commonly, this is the exception, because crystal growth processes and the presence of extra-lattice particulates yields trace element distribution inhomogeneities along the same seasonal/annual layer. Chronologies built upon annual trace element cycles that can be precisely compared with the physical aspects of layering in speleothems are the most reliable, because peaks and troughs along sampling transects at high spatial resolution can be visually superimposed to fabrics^16,17,23,24^. However, annual laminae-specific heterogeneities due to crystal fabrics and presence of nano-particulate can only be detected by two-dimensional, continuous, high resolution trace element mapping^25^, which can be performed by using Synchrotron radiation-based micro X-Ray fluorescence over entire speleothem slabs^26,27^.

Speleothems from regions with high seasonal contrast typically show annual cyclicity in trace element concentration of bedrock-derived solutes (such as Mg, Sr, Ba, U)^13,14,18,28^, soil-derived particles (such as P, Al, Fe, U) and of those elements whose partitioning depends on growth rate (P, Al, Sr). The assumption in using bedrock-derived trace element cycles for dating stalagmites is that they follow cave ventilation and/or prior calcite precipitation (PCP: calcite precipitation from a solution before it reaches the stalagmite)^29^ and/or changes in rock-water interaction (RWI) processes, which are all related to temperature and/ or rainfall contrast. In Mediterranean and monsoon climates, it stands to reason that during the dry season the Mg/Ca and Sr/Ca ratios in the parent water and, thus, in the speleothem carbonate are expected to increase^30^. Yet, there are other processes contributing to high-frequency variability in Mg and Sr concentration within speleothems, such as enhanced wind-blown delivery of marine aerosols to cave sites proximal to the coast^31,32^. Another source of Sr could be dust-blown particulates, although this provenance for Sr seems to be linked to large-scale, secular-to-millennial climate changes^33–35^. Thus, even Mg and Sr could be potentially incorporated into speleothem layers as particulate and produce anomalous (outliers) peaks in their concentration. An increase in concentration of P could be related to the development of microbial films on top of stalagmites during dry periods, as well as soil provenance in wet periods, particularly in tropical island settings^36^. Thus, P may not be taken as a universal “wet” conditions marker, as this assumption is valid for stalagmites grown under conditions associated with soil and vegetation decay in monsoon-dominated or Mediterranean climate settings. For the tropical South Pacific, where maritime climate conditions prevail, the interpretation of P as climate proxy is still controversial. A nano-scale investigation of carbonate crystal growth in cave highlighted the possibility that this occurs by particle attachment, where particles are amorphous phases whose incorporation of Sr, Mg, Al, Fe and P is still poorly known^26^. It is, therefore, desirable to build a tropical South Pacific speleothem chronology by considering seasonal variations in elements, such as Yttrium, which are known to be particulate-bound, soil-derived and transported colloidally during high infiltration events^17^. Seasonal variation in Y are, likely, a most robust record of seasonal infiltration in tropical settings.

There is a need to overcome U–Th age uncertainties with a methodology that can be applied to all tropical, low seasonality contrast stalagmites^8^. Here, we propose to apply a two-dimensional (2D) approach to obtain an optimized chronology for a tropical, low-seasonal contrast South Pacific stalagmite with high U–Th dating uncertainties based on coupled visible and trace element concentration layers. This was achieved by coupling high-resolution imaging of fine-scale fabric variability and their associated 2D synchrotron-radiation based micro X-ray fluorescence (SR-µXRF) mapping of trace element concentrations. The high sensitivity and spatial resolution of SR-µXRF make it a superior technique over conventional µXRF (e.g. benchtop µXRF^37^), particularly when annual to sub-annual laminae thicknesses are less than 100 µm. We also generated one-dimensional (1D) transects for Mg, Sr, Na, U, Ba, P and Y cycles by using the most common technique applied in palaeoclimate research, laser ablation inductively coupled plasma-mass spectrometry (LA-ICP-MS), to highlight the differences between a 1D and a 2D mapping approach.

Following the protocol outlined in Nagra et al.^13^ we employed principal component analysis (PCA) to construct a robust chronology, and then we developed an aridity index to constrain inter-annual variability. The variability of the aridity index was then synchronized with the annual record of local hydroclimate variability and the Southern Oscillation Index (the difference between the sea level pressure at Tahiti and Darwin, which is an atmospheric see-saw that straddles our site). This combined approach resulted in an age model with only 4% uncertainty, considerably advancing the ca. 50% uncertainty in the U–Th age model. Our findings imply that reliable annual hydrological variability can be obtained from “problematic” tropical samples where U-Th dating is difficult even when seasonal contrast is not strong.

## Building chronology for stalagmite Pu17

Below we explicate the methodological procedure to construct an accurate chronology for stalagmites from low seasonal contrast tropical Pacific climate using Sr 2D maps, fabrics and annual hydrological cycles.

### Site description and stalagmite sample

Pouatea cave (20° 01′ 12″ S, 158° 07′ 10″ W), is located in Atiu, the third largest island of the southern Cook Islands (SCI) archipelago (Supplementary information, Fig S-1). Atiu is geologically characterized by a core of weathered and eroded basaltic rocks rimmed by an uplifted Pliocene–Pleistocene reef limestone (known as “matakea”)^38^ (Supplementary information, Fig S-1). The makatea hosts a maze of almost horizontal solution cave passages, with lower levels that reach the water table and discharge freshwater into the ocean^39^. Atiu is characterized by a relatively small annual temperature range from 22 °C in winter up to 26 °C in summer. Rainfall is influenced by seasonal shifts of the South Pacific Convergence Zone (SPCZ)^40–42^, tropical cyclones (TC)^43^ and, on decadal timescales, by the effects of ENSO^44^. Southwest deflection of the SPCZ over Atiu results in unstable weather from beginning of November to end of April (aka ‘the wet season’) when ~ 60% of the total annual rainfall (TAR = 1930 ± 365 mm/year) occurs and when the mean monthly rainfall varies from ~ 150 to 200 mm. The “dry” season, when mean monthly rainfall is ~ 50 to 100 mm, occurs from May to October when the SPCZ moves northward and is less organised at the same time when prevailing flow is dominated by south easterly trade winds^38,45,46^. Local records of monthly precipitation available for the SCI stations at Mangaia (Jan 1914–May 1996), Aitutaki (Jan 1930–Jun 1996), Rarotonga (Jun 1948–Feb 2016) and Atiu (Oct 1980–Mar 2019) show consistency in the precipitation data where records overlap, suggesting that Atiu is representative of the mean climate conditions and variability experienced across the southern Cook archipelago (Supplementary information Fig S-2).

Pouatea is a network cave system that has several intersecting and side galleries with a total surveyed length of ~ 1200 m. The main entrance of the cave opens 23 m above sea level at 525 m from the shoreline and is a vertical shaft with a drop of about 4 m. In addition, there are five other entrances (skylights) with diameters ranging from 3 to 10 m formed as a result of cave roof collapse. The rock burden above the galleries is 4 to 8 m, due to incomplete diagenesis of a relatively young reef, characterized by high porosity. This ensures rapid transmission of surface climate conditions into the cave, which is further enhanced by a limited and patchy soil cover. Except for some small pockets of red clay soil filling the bottom of joint-controlled karst of colluvial origin^38^, the surface above the cave is a bare karst. Trees push their roots through holes and fractures in the makatea all the way to the cavern system to source moisture. Most of the visible organic matter in the makatea consists of leaves, nuts and bark, all of which are rapidly degraded under the warm and wet climate conditions of the island. Because of the sparse soil cover and barren karst, the infiltration area is bound to be subject to evaporation during the relatively dry and warm summer months (Dec, Jan, and Feb).

Stalagmite Pu17 was actively growing when removed in March 2019 at a depth of ca. 7 m beneath the surface within a gallery leading to the southern dead-end of the cave. The feeding straw was still dripping at the time of retrieval and a thin calcite layer formed on the stump of the removed Pu17 stalagmite between March and October 2019, corroborating the idea that the top of Pu17 has a date of 2019 CE. Pu17 was fed by a relatively slow and constant drip (1 drop each 15 min), which resulted in its candle-shaped morphology^47^. Pu17 is 53 mm long and grew over a stalagmite stump, likely broken by humans before European colonization, according to Atiuan lore, which highlights the importance of speleothems as ceremonial building material^48^. The stalagmite then provides an opportunity to unravel a history of Polynesian land-use as well as climate variability.

Cave monitoring data are available from 2017 to 2019, although the remoteness of the site allowed four visits, only one of which in the wetter season. Mean air temperature in the passage where Pu 17 was collected is 23.6 ± 0.5 °C with minimum values in September–October and maxima in March–April. Cave air pCO_2_ varied from 500 and 650 ppm in the relatively dry and wet seasons respectively, while relative humidity was at near saturation (98–100%) throughout the year. Continuous drip rate monitoring at two drip points compared with daily precipitation data suggests that there is fast transmission of the rainfall signal into the cave, with time lag of less than two days between intense rainfall events and maximum values in drip rates (Supplementary information, Fig S-3). Drip rate monitoring is also indicative of a relatively dry period between May to October characterised with a gradual decrease in the rate of dripping. The pH of the dripwaters in the cave is ~ 8 and the Saturation Index for calcite (SIcc) varies from 0.9 to 1. Calcite that was formed in situ on watch glass under relatively fast drip rates (~ 1 drop/11 s), shows organic and inorganic colloids (mostly oxihydrates of Fe, Al, and Si) adhering to the crystal surfaces and/or settling between rhombohedra (Supplementary information, Fig S-4). This results in a more porous fabric compared to compact fabrics developed under slow dripping feeding systems. Under relatively slow drips (1 drop/50 s) the crystals start to coalesce and crystal boundaries interlock, leading to a translucent and compact fabric (Supplementary information, Fig S-4). In-situ precipitation experiments suggest that low drip rates (equivalent to a prolonged residence time of the thin film of fluid atop a stalagmite) result in an increase of Mg in the precipitates accompanied by the formation of NaCl crystals, which suggests contamination by marine aerosol (Supplementary information, Fig S-5).

The U content in Pu17 varies between 80 and 130 ppb, which is higher than other active and modern stalagmites from Pouatea cave. A most likely initial ^230^Th/^232^Th activity ratio of 6.12 ± 0.84 was determined after the exclusion of two outliers using the stratigraphic constraint procedure^49^ and an age-depth model was determined from the remaining eight analyses using the finite positive growth rate model^50^ and the assumption of active growth at time of collection. The radiometric U–Th ages have large 2-s uncertainties (Supplementary information, Table S-1). Given that pre-instrumental annual rainfall data for Atiu would be fundamental to test the reliability of predictive climate models, building an annually resolved chronology with physical and chemical cycles modulated by a muted seasonal contrast becomes a necessity.

### Two-dimensional-based lamina counting

**Figure 1:**
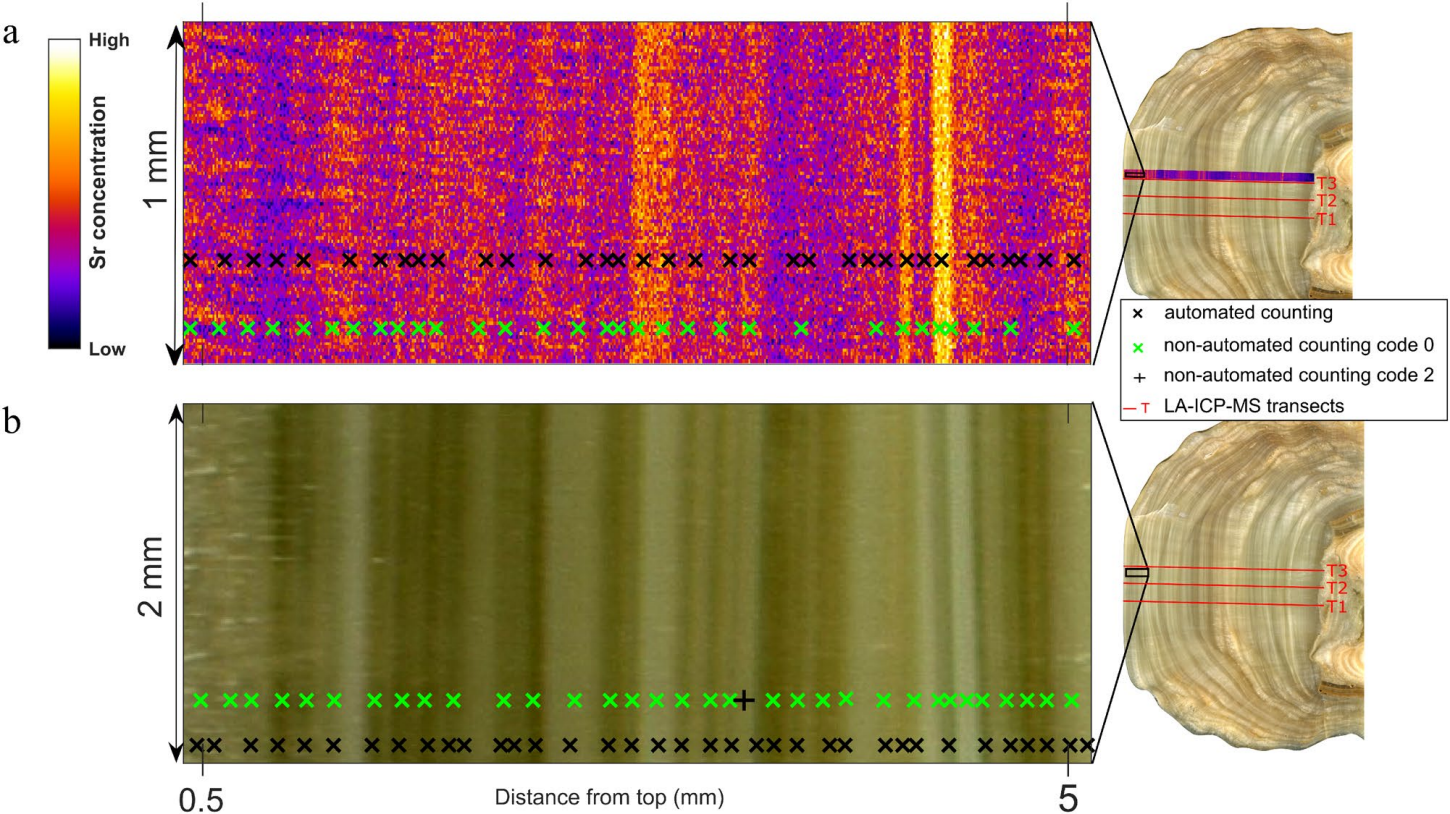
Locations of laminae on (**a**) the Sr synchrotron µXRF map, and (**b**) the high resolution scan of stalagmite, as identified by automated and non-automated counting in the topmost section of the stalagmite.

An annually-resolved chronology can be constructed from fabrics and chemical laminae seen in two dimensions. The physical laminae were here highlighted by using a high-resolution scanner to identify variations in compact and porous fabrics, which are related to infiltration due to the inhibiting effects of particulate on crystal growth processes during higher infiltration (see previous section). 2D trace element (geochemical) laminae were visualized by 5 μm resolution SR-μXRF maps and highlight spatial (time-equivalent) homogeneity and/or heterogeneity of trace element distributions within physically visible growth laminae. Trace element peak shifts recorded by 1D parallel LA-ICP-MS were superimposed to fabric maps and shifts in TE concentration could be explained by lateral heterogeneities. These were highlighted by the SR-μXRF 2D maps, which were thus taken as reference to anchor the chemical cycles detected by LA-ICP-MS scans and then obtain a direct comparison with physical laminae (see Fig. 1). Of all the trace elements detected by SR-μXRF (Fe, Br, and Sr), only Sr shows cycles of concentration variability that can be followed along physical growth laminae, with cycles spaced at ∼ 50 to 150 μm intervals. The increase in Sr concentration is not unequivocally correlated to a compact or porous fabric (Fig. 1), yet the 2D map highlights that there exist sharp boundaries for Sr-concentration laminae. The position of the onset of Sr laminae (cycles) in the SR-μXRF maps was then visually determined in a non-automated approach at three levels of certainty through the ImageJ software package. The advantage of SR-μXRF 2D mapping is that it allows for both an evaluation of the lateral chemical prominence and persistence of trace elements within each lamina and the identification of outliers due to particulate inclusions.

In our 2D approach, prominent and persistent Sr laminae have been given, for convenience, the code 0, which applies to Sr laminae characterized by > 95% confidence about their probability of occurrence. Code 1 was applied to persistent laminae with low prominence and more than 50% probability of occurrence. These laminae are laterally persistent but of low or very weak prominence. Examples of this group are sub-annual laminae that show feeble prominence but can be followed laterally. Code 2 is used for locally prominent laminae showing feeble lateral persistence (less than 50% probability of occurrence). This group includes laminae that cannot be followed laterally in the 2D Sr map. By accounting for the probability rating of geochemical laminae, group 0, 1 and 2 were integrated in the age model with ± error of 0, 27 and 72% respectively. The error was calculated from the probability of occurrence, such that, as an example, code 1 with 50–95% probability would have an error equal to $\frac{100-\left(\frac{50+95}{2}\right)}{100}$.

The visual method may carry an inherent error due to the operator’s discretion. Thus, trace element concentration laminae in the SR-μXRF Sr map were independently and automatically identified by using the Open Computer Vision Library in the Python programming language. A 170 pixels wide (0.85 mm) scan was extracted along the Pu17 growth axis using an Adaptive Gaussian Thresholding. This approach minimised lateral heterogeneity along each growth lamina and is similar to the visual evaluation by encompassing the entire width of the map, provided that laminae are perpendicular to the vertical growth extension. Thresholding is the simplest segmentation method that separates regions of an image corresponding to chemical laminae that are the target of the analysis. Here, the separation was based on the variation of intensity between the pixels associated with Sr-rich laminae and the background pixels. In order to increase the accuracy of the link between automated and non-automated results of laminae positions with the geochemical cycles obtained in the LA-ICP-MS transects, a line scan was extracted from the SR-μXRF Sr map adjacent to a LA-ICP-MS transect (see Fig. 1). The extracted SR-μXRF Sr line scan, was smoothed using a Savitzsky-Golay filter (order of 9 and window length of 31) and peaks corresponding to Sr-enriched layers on the SR-μXRF Sr map were then counted using the Python *find_peaks* command. The find-peaks function takes the line scan as the input signal vector, and pixel index as the location vector and returns the value, location, and prominence for each peak. Minor and/or double laminae due to inter-annual events can thus be eliminated by applying minimum distance and minimum peak prominence constraints. These are two optional adjustments that enable the user to control peak counting. For example, by applying a minimum peak distance of 10 pixels (50 µm), *find_peaks* identifies and isolates the highest peak in the signal and eliminates all peaks within 0.05 mm around it. The function then repeats the procedure for the highest remaining peak and iterates until it reaches the last peak of the signal. A minimum peak prominence of 10 ppm, for example, means that a peak is counted only if it drops 10 ppm on either side of its maximum. The number of significant layers was determined by applying a peak distance of 50 µm (~ half of the distance determined visually) and matching peak prominence with visual laminae.

Manual (non-automated) and automated lamina counting was also carried out on a 10 μm resolution image of physical growth between LA-ICP-MS transects 2 and 3 (T2 and T3 in Fig. 1), and adjacent to the area imaged by SR-μXRF (see Fig. 1). The visible physical laminae in Pu17 results from alternation (couplets) due to changes in micro-porosity or particulate concentration of the calcite layers, which yields different optical properties typical of textural laminae as defined by (Genty and Quinif^51^). Translucent, optically dark layers are compact, whilst the opaque, optically whitish layers are porous and characterized by the presence of intercrystalline particulate, which is more efficient in inhibiting calcite crystal growth when drip rate increases (cf.^13,20,51,52^). The position and number of translucent bands (lower drip rates) were determined at three levels of certainty (similar to Sr 2D map) by using ImageJ software. Although the optical scan of Pu17 fabrics has a lower resolution (10 µm) compared to the SR-µXRF map (5 µm), it captures growth surfaces in two dimensions and thus enables to assess the prominence and persistency of growth bands in a broader area. The translucent layers were also counted automatically with *find_peaks* by using the same peak distance applied for the SR-µXRF generated Sr concentration 2D map. The automated counting of translucent bands was conducted on a line scan extracted from the Pu17 fabrics grey-scale image. Translucent fabrics (darker bands) are characterised by higher grey values. Peak prominence was then optimized to match the number of peaks (translucent bands) with the laminations identified with the visual counting.

### Counting cycles on one-dimensional LA-ICP-MS transects

For counting number of cycles on LA-ICP-MS transects, we selected three transects in the central growth section of Pu17 (T1, T2 and T3 in Fig. 1). Mg, Sr, Ba, Na, U, P and Y show clear cyclic signal. In order to determine the annual nature of geochemical cycles and build an annually-resolved chronology for South Pacific speleothems, the following procedure was followed:

1. a Savitzky-Golay filter of the order of 9 and window length of 31 was applied to remove high frequency noise.
2. individual elements concentration was standardised by subtracting the mean and dividing by standard deviation.
3. in order to improve the consistency between three transects, individual elements on each transect were resampled at 6 μm resolution.
4. implementation of principal component analysis (PCA) on smoothed, scaled and resampled elements in each transect in order to find the maximum correlation between different elements and provide two principal components that account for most of the variance. PCA-A includes the six hydrologically sensitive elements Mg, Sr, Ba, Na, P and Y, while PCA-B was restricted to four elements (Mg, Sr, Ba, Na) sensitive to bedrock-related processes (PCP/RWI). U was excluded from PCA-A because it has a very noisy signal.
5. finding the maximum number of prominent peaks by applying *find_peaks* on the individual elements and the principal components.

The master chronology was built by integrating all lamina and trace element cycle counting methods and assigning calendar ages to the layer counts by working from the date of collection backwards. For around 80% of the record in each lamina couplet the compact, translucent layer marking a relatively low drip-rate coincides with higher Sr and Mg concentrations than the more porous layers^13,16^. By assuming that the optically dark, compact and the optically whitish, more porous layers identify changes in drip rate, supersaturation and efficiency of growth inhibitors as modulated by changes in rainfall, we tested the robustness of our assumption that laminae are annual against instrumental rainfall record and SOI. The internal speleothem chronology was then optimised by achieving the best match between Pu17 infiltration proxies and the SOI. This implied that we had to transform rainfall data into effective infiltration, which modulates the drip rate. Potential evapotranspiration (PET) was first calculated for Mangaia, Aitutaki, Rarotonga, and Atiu by using the Thornthwaite equation^53^, and then the effective infiltration amount was obtained by subtracting PET from the rainfall amount. The infiltration values of Mangaia, Aitutaki, and Rarotonga were averaged for the portion of the record where they overlap and tied with Atiu’s record to obtain a continuous infiltration series from January 1914 to March 2019.

### Validation of Pu17 chronology: regression and wavelet analysis

In order to further evaluate the accuracy of the our age model, the infiltration record from Atiu was modelled via inputting a group of trace elements (Mg, Sr, Ba, Na, U, P and Y) into a Gaussian Process Regression (GPR), a nonparametric kernel-based probabilistic model^54^. The GPR in this study is implemented in MATLAB software by using the regression learner module. Prior to running the GPR, concentration signals of trace elements were detrended to obtain a fully stationary signal. Detrended signals of trace elements were achieved after identifying 10 most abrupt changes from the root-mean-square level of the trace elements concentration, and then linearly removing these abrupt changes from concentration values. Since detrending removes components of the trace elements signal that are associated to the effects of decadal to multi-decadal climate drivers oscillations, this process must be exercised with caution when trace elements are used to extract annually resolved past-climate information. By detrending, we aimed to obtain a stationary dataset for the sole estimation of infiltration, given that the reliability of the constructed chronology is based on the assumption that subtle annual hydrological changes occur. Thereafter, the normalised and detrended time series of trace elements for the interval of 1914–2019 were split into two training and test sets, with the latter being the 30 year-long period from 1914 to 1944. The GPR model was applied on the training set with a tenfold cross-validation configuration. This validation protects against overfitting by partitioning the dataset into batches (folds) and then estimating the accuracy on each batch. The effective infiltration was estimated by applying the GP regression to the test data (final validation) and then to the whole stalagmite. The results of the GPR model on the test data were evaluated by comparison with records of calculated infiltration (acquired via Thornthwaite equation) and SOI. We used LA-ICP-MS transect-2 (see Fig. 1) to develop the GPR model because, by anchoring the transects to 2D maps, it was reasonable to assume that line scans are representative of stalagmite layering. The reliability and accuracy of our modelled infiltration for the test data period (1914–1944) was finally evaluated through wavelet coherence analysis and the results compared to SOI data to check for the robustness of our chronology. The coherence was computed using the Morlet wavelet in MATLAB software and *wcoherence* function.

## Results and discussion

The three independent counting techniques produced 37 series, 20 of which were then averaged and integrated into an age model. The mean growth rate calculated from the U/Th age model (139 ± 39 μm/year) was found to be similar to that calculated from lamina counting (144 ± 7 μm/year).

### Building the chronology from lamina and geochemical cycles

**Figure 2:**
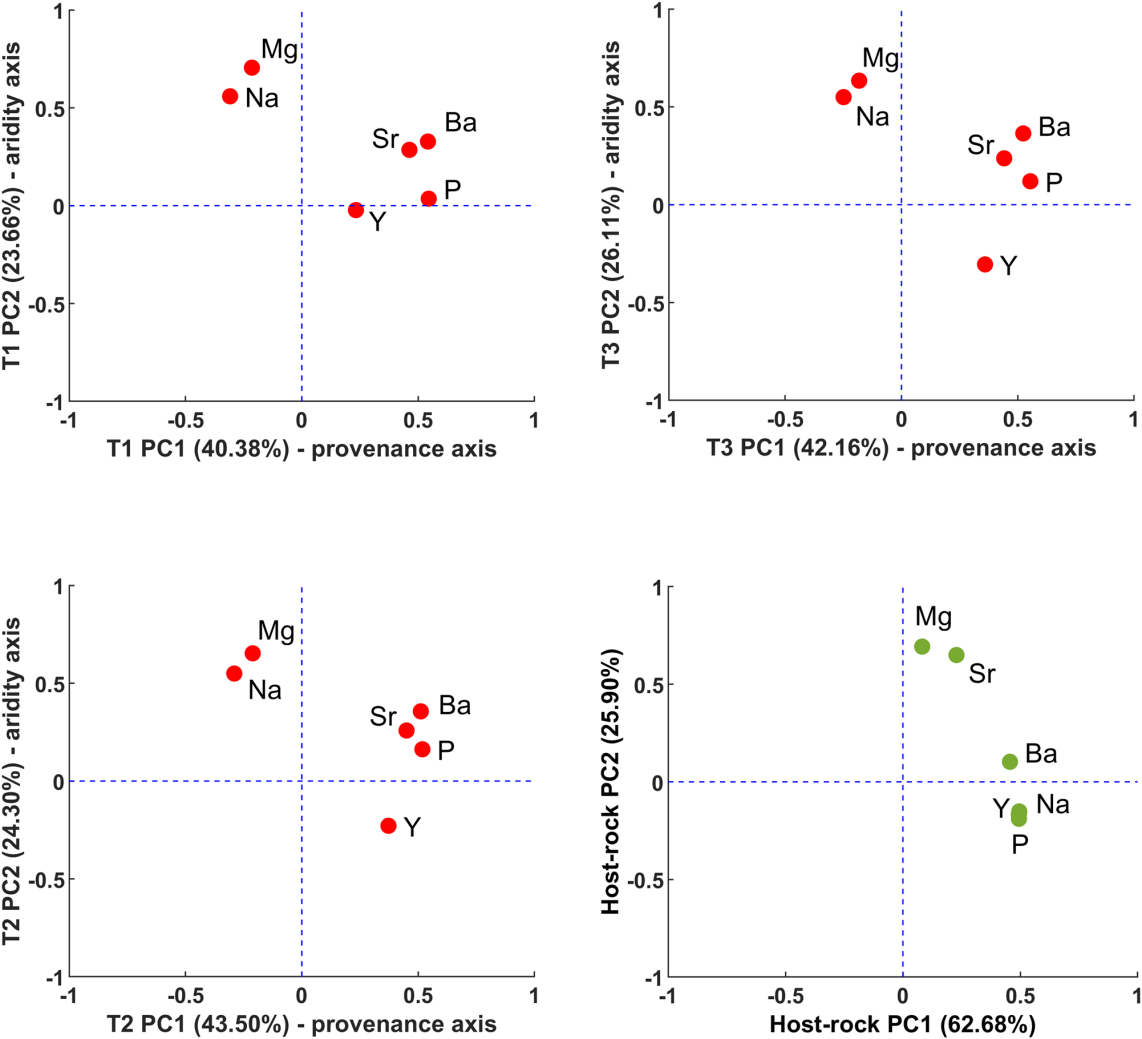
Loadings of different elements in PCA-A on stalagmite trace elements measured at three transects (plots with red markers), and on host-rock trace elements (plot with green markers).

Pu17 microstratigraphy, consisting of high-frequency alternation of translucent compact (yellow–brownish) and opaque porous (whitish) low-Mg calcite layers stacked along the vertical growth axis (see Fig. 1) likely results from the interplay between SIcc, pH, particulate load, changes in Ion Activity Product due to the effects of marine aerosols, and growth mechanisms. The complexity of these processes demands an understanding of the provenance of trace elements, which can be obtained through PCA. We employed two principal component analyses: PCA-A on [Mg, Sr, Na, Ba, P, Y] and PCA-B on [Mg, Sr, Na, Ba], following identification of laminae in both the SR-μXRF Sr maps and visual scan (Fig. 1). Two main principal components for each PCA in each transect were obtained, which accounted for much of the variance in trace element concentration distributions (Supplementary information, Table S-2). Figure 2 shows the loading of different elements in PC1 and PC2 of PCA-A. PC1 of PCA-A explains 42 ± 1.5% of the trace element variability in all three LA-ICP-MS transects (Table 1). The elements Sr, Ba, P and Y have positive values for PC1. Of these, Sr and Ba most likely originate from the host rock and their similar behaviour and strong, positive correlation is well documented for speleothems^28,55^. By contrast, Y appears to be a soil-derived element^56^, similar to P, although P has a complex behaviour^36^ ^and^ ^references^ ^therein^. In Pu17, P plots on the positive side of PC1, but bears more affinity with Y than Sr and Mg in PC2 axis. The origin of P concentrations in tropical South Pacific islands appears to be related to weathering of corals^57^, which would explain its association in PCA with both soil- and rock-derived elements. The elements Mg and Na plot on the negative side of PC1. Mg is commonly believed to derive from the dissolution of a carbonate bedrock^58^, however, its separation from bedrock-derived elements in PC1, and its plotting with Na suggests a predominant contribution from sea-spray and/or any surface source. The sea-spray source is a reasonable hypothesis, considering that Pouatea is 0.5 km from the coast and marine aerosols easily reach the cave site, particularly in windy and rough sea conditions as documented for coastal caves elsewhere^59^. The PC1 axis then is a “provenance axis” with the positive side related to host rock and soil sources and the negative to marine aerosols and/or products of coral reef weathering.

**Table 1:** The results of counting on a group of trace elements, principal components, and synchrotron and petrography images.

Proxy	Transect-1		Transect-2		Transect-3	
	Minimum peak prominence ± 5%	Number of peaks	Minimum peak prominence ± 5%	Number of peaks	Minimum peak prominence ± 5%	Number of peaks/laminations
Mg	0.19 ± 0.009	322 ± 9	0.137 ± 0.007	341 ± 3	0.145 ± 0.007	334 ± 5
Sr	0.228 ± 0.011	325 ± 11	0.22 ± 0.011	345 ± 8	0.23 ± 0.011	337 ± 9
Ba	0.58 ± 0.029	314 ± 22	0.54 ± 0.027	325 ± 20	0.58 ± 0.029	306 ± 23
Na	0.133 ± 0.006	358 ± 2	0.16 ± 0.008	341 ± 5	0.229 ± 0.011	329 ± 12
PCA-A PC1	0.28 ± 0.014	326 ± 3	0.29 ± 0.014	332 ± 12	0.296 ± 0.014	332 ± 8
PCA-A PC2	0.37 ± 0.018	331 ± 12	0.38 ± 0.019	328 ± 9	0.422 ± 0.021	330 ± 12
PCA-B PC1	0.33 ± 0.016	341 ± 10	0.32 ± 0.016	339 ± 9	0.37 ± 0.018	339 ± 10
PCA-B PC2	0.273 ± 0.013	339 ± 5	0.24 ± 0.012	349 ± 7	0.26 ± 0.013	332 ± 6
Synchrotron Sr map	–	–	–		19 ± 1	330 (auto)
					–	335 (non-auto)
Visual scan	–	–	–		51 ± 3	335 (auto)
					–	329 (non-auto)
Average	–	332 ± 9	–	338 ± 9	–	335 ± 11

In the PC2 of PCA-A, which accounts for 24.8 ± 1.2% of the variance, all the elements plot positively except for Y, which is clearly the only element whose provenance is exclusively from the soil and is carried into the cave as adsorbed species on particles^56^. Thus, Y shows negative peaks (decrease in concentration) during periods of low infiltration and positive peaks (increase in concentration) during higher infiltration. Because P could also be sourced from the soil (as a product of weathering of the rock), its behaviour is intermediate between that of Y and [Sr, Ba]. We, therefore, argue that PCA identifies infiltration as the mechanism controlling the concentration of trace elements in Pu17 at a seasonal scale, regardless of the trace element provenance. Higher infiltration (i.e. higher rainfall at a relatively constant temperature) corresponds to less RWI, less PCP and less efficiency of marine aerosol-derived elements in influencing calcite growth processes. Thus, when drip rate is relatively high, we would expect the formation of calcite layers with a more porous fabric and relatively low Mg, Na, Sr, Ba concentrations. By contrast, in relatively wet periods, soil-derived elements, such as Y (and, in part, P), would increase. The PC2 axis is the “relatively dry” axis for Atiu, where Mg, Na, Sr, Ba and P plot positively and Y plots negatively. The more positive values of PC2 correspond to enhanced PCP/RWI as a result of lower infiltration, and its negative values coincide with low PCP/RWI and higher infiltration. LA-ICP-MS analysis of host rock sample above Pouatea cave corroborates the idea that Mg concentration in Pu17 has two sources. PCA of trace elements in the rock shows that they all plot on the positive side of PC1 and on PC2, Mg and Sr behave similarly (see Fig. 2), which suggests that marine aerosol is a likely additional source of Mg incorporated in stalagmite calcite^59^.

**Figure 3:**
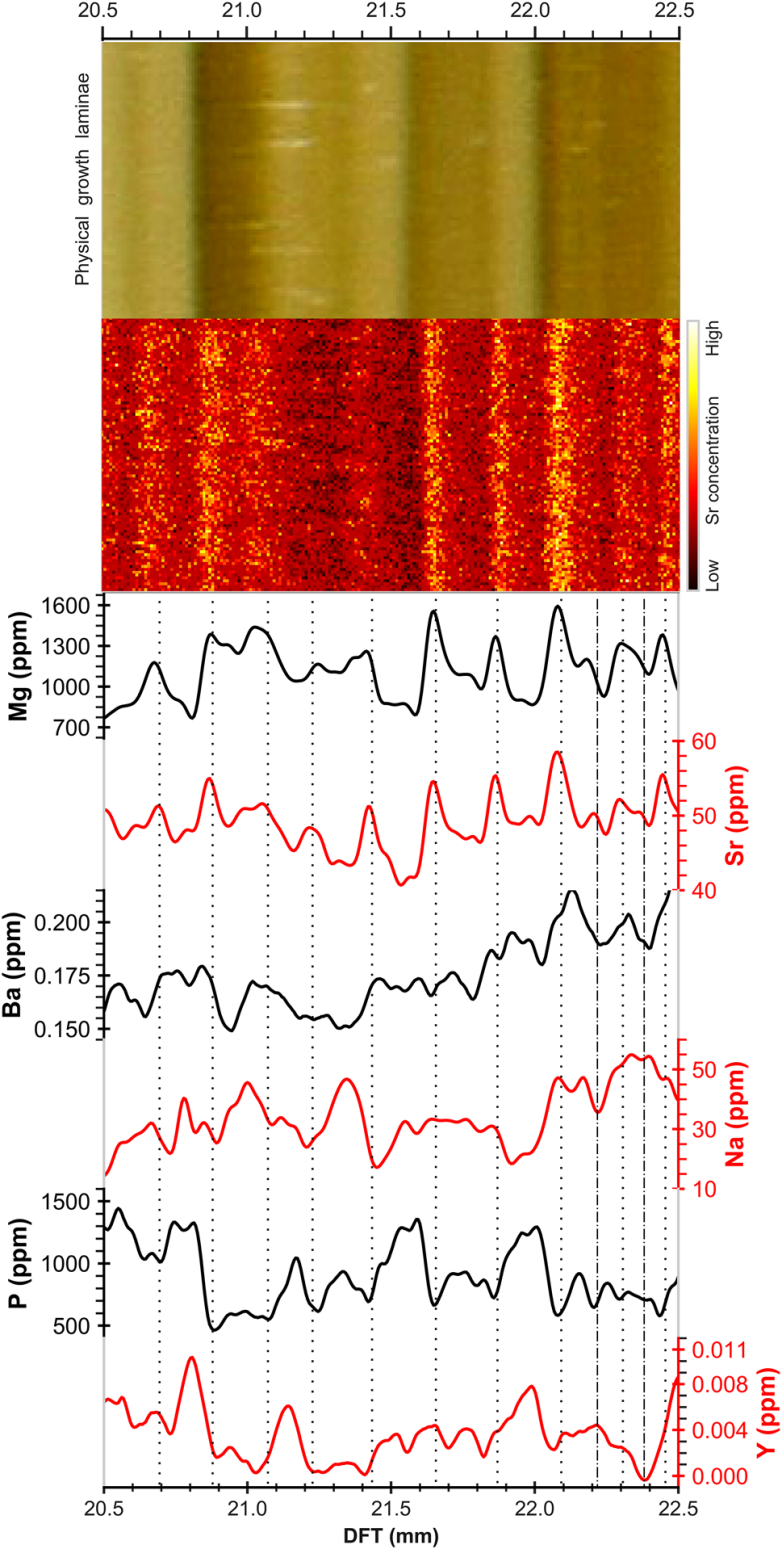
Annually laminated section of Pu17 between 20.5 and 22.5 mm from the top. The dotted vertical lines mark likely annual cycles identified by maximum Sr values on the synchrotron map. These generally correspond to maximum values for Mg, Sr, Ba, Na, U and minima for P and Y on the LA-ICP-MS record. The dashed lines are dubious cycles identified by peaks in only few elements (sub- or double-laminae). Translucent laminae on the physical sample usually correspond to Sr-enriched laminae on the synchrotron map.

Figure 3 shows that the elements plotting on the positive side of the “aridity axis”, i.e. Mg, Na, Sr, and Ba show similar cyclicity and their concentration commonly (but not always) increases in compact laminae (Fig. 3), here interpreted as formed during relatively low infiltration. It is likely that PCP and/or RWI modulate their cyclicity. By contrast, P and Y show positive concentration peaks in porous, opaque calcite, here interpreted as a marker of higher drip rate (wet phases), as they are preferentially transported from soil to stalagmite surface as organic/inorganic particulates during higher infiltration conditions^17^.

**Figure 4:**
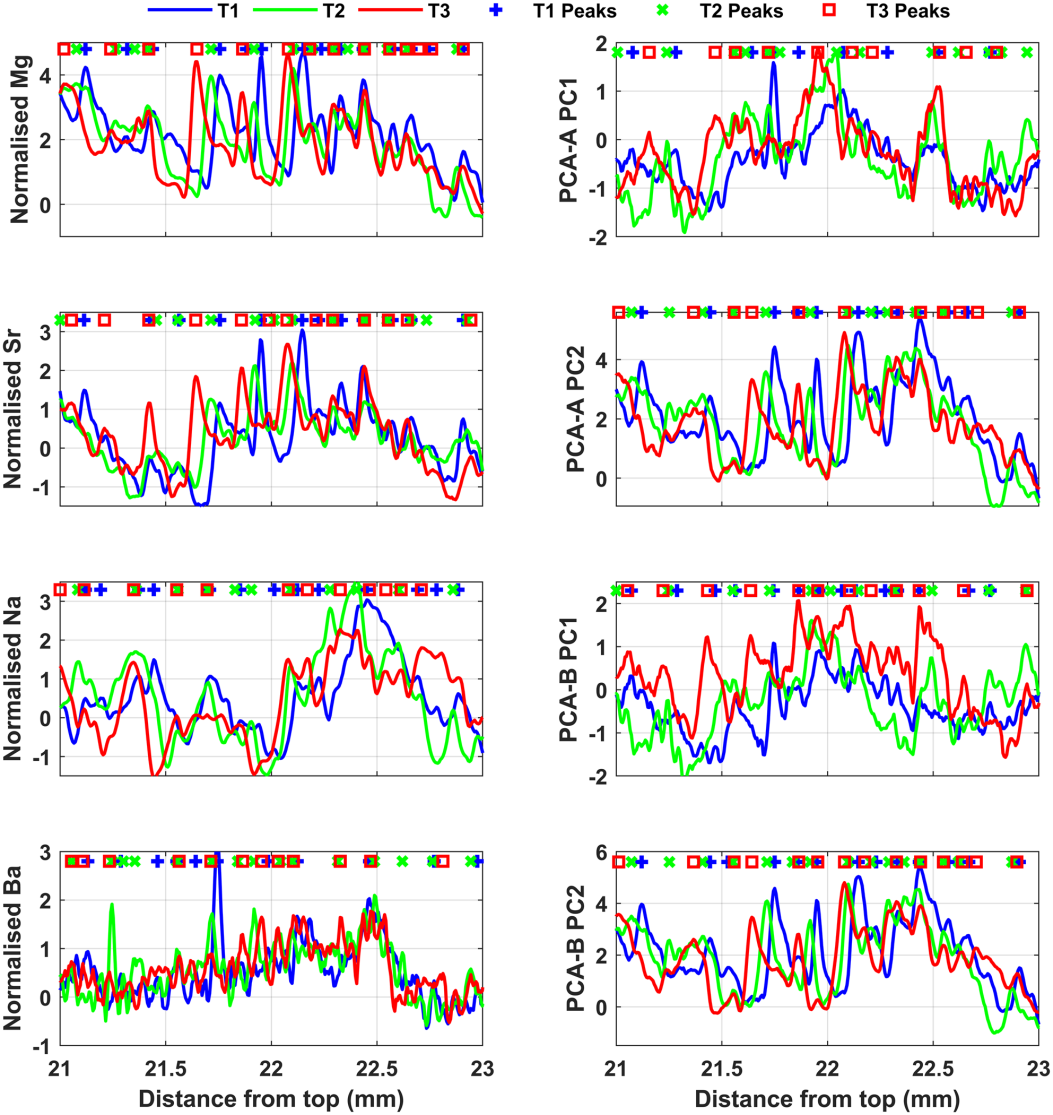
The locations of peaks identified on a group of LA-ICP-MS trace elements and the principal components. PCA-A includes [Mg, Sr, Na, Ba, P, Y] and PCA-B contains only [Mg, Sr, Na, Ba].

PCA-B was established by focusing the analysis to elements whose behaviour is similarly affected by infiltration (Mg, Na, Sr, Ba). The PC1 and PC2 of PCA-B explain 42 ± 1% and 35.5 ± 1% of the variance, respectively. In PC1, Sr and Ba load strongly and with positive values, whereas Mg and Na load negatively. All the four elements plot on the positive side of the PC2 (Supplementary information, Table S-2). The hydrogeologic significance of trace elements was established through PCA, consequently, we identified that trace element cycles define subtle annual changes in infiltration in a stalagmite grown under low seasonal contrast conditions. We then applied *find_peaks* to count calendar years by identifying annual cycles in trace element concentration. The locations of peaks in the “relatively dry” trace elements group including Mg, Na, Sr, and Ba and the principal components are shown in Fig. 4. PCA reinforces peak counting because it explains much of the variability observed in a trace elements’ concentration. The chronology based on principal components of a group of elements is more inclusive than that based on single elements and this minimises the effect of missing peaks that may occur in single element concentration analysis. However, as shown in Figs. 3 and 4, in some parts of the record the annual variations of trace elements is ambiguous and cycles are not clear. In order to tackle this problem Sr 2D maps were taken as a reference to anchor the chemical cycles detected by LA-ICP-MS scans. In addition to 2D maps, the advantageous of using a group of trace elements is that if a cycle is repeated in most of the proxies, it is more probable to be an annual hydrological cycle, even though of feeble prominence.

The minimum peak prominence adjusted for a group of elements in LA-ICP-MS T1, T2 and T3, and number of identified peaks are listed in Table 1. A ± 5% error is incorporated in peak prominences and in the number of counted peaks. Table 1 also includes the results of laminae counting in the SR μXRF Sr map, and in the fabric scan of Pu17, determined by automated and non-automated methods.

**Figure 5:**
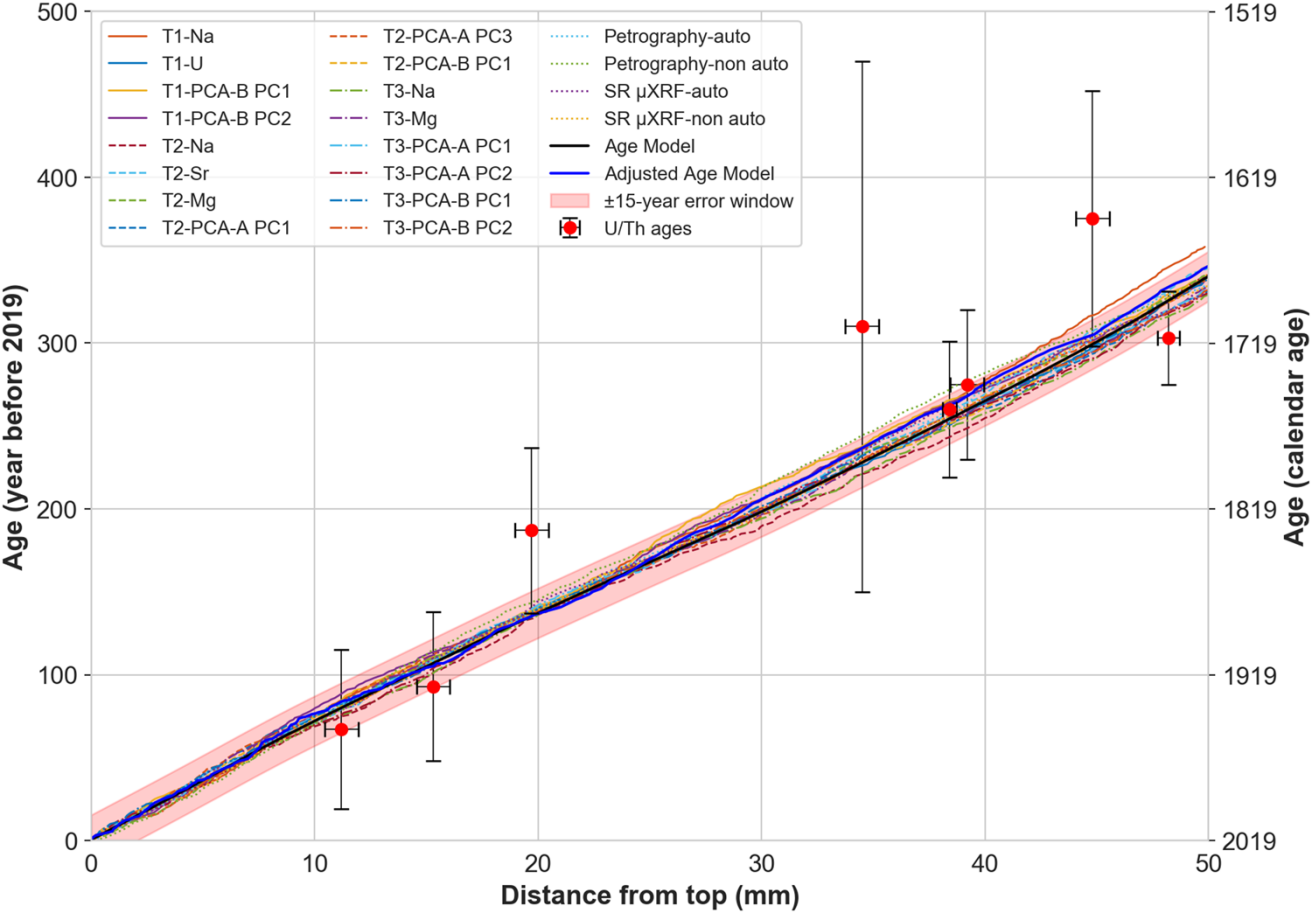
Selected time-depth curves, calculated (solid black) and adjusted (solid blue) master curves. The ± 15-year error window (red band) envelopes all the selected curves for most of the age interval. The red circles and vertical capped lines demonstrate the U/Th ages and associated uncertainties.

Having established that trace elements follow hydrological cycles, we here infer that trace element concentration variability follows annual cycles, although subdued relative to Mediterranean and Monsoonal settings. Counted chemical and physical cycles allowed the creation of time-depth (TD) curves along each LA-ICP-MS transect and 2D mapping for all hydrological proxies, which include individual trace elements, principal components, fabrics, Sr concentration distribution along a growth lamina and the associated fabric. We obtained a total of 37 curves for all transects. The number of peaks/laminations varies in different proxy-data series, however, 20 curves have a mean of 336 ± 15 laminae. These 20 curves were then averaged and integrated into a single master chronology where the number of laminae equals 336 years. Figure 5 shows selected TD curves and a corresponding average curve (solid black). Averaging further assists to exclude from the master chronology those laminae/cycles which are not prominent and have probably appeared in one or a couple of proxies. The master chronology was slightly adjusted by synchronising significant peaks/troughs in Mg concentration (from T2) to construct the final age model. This was accomplished by stretching/squeezing the age model, within the ± 15 uncertainty limits, to match similar patterns between Mg and local infiltration and SOI records. Mg was used to adjust the age model because compared to other elements it has a better correlation with local infiltration. That is in line with our finding that, although Mg has multiple sources, its concentration in speleothem is controlled by effective infiltration via PCP/RWI processes. The chronology of the stalagmite growth was then extended by 11 years (3% change), so that the final age model (shown in Fig. 5 by solid blue curve), indicates that Pu17 grew in the last 347 years. The final age model has a ± 15-year error window (red band) that envelopes most of the curves selected to build the master chronology. The discrepancy between initial and adjusted master chronologies for the top 25 mm of the stalagmite is less than 8 years and averages 2–3 years. For the bottom 25 mm, the maximum difference is 10 years. Most of the curves are characterized by a diverging pattern at the bottom half of Pu17, probably because the laminations are less clear (Supplementary information, Fig S-6). At this interval the adjusted curve follows the trend of the SR-μXRF Sr maps and fabric curves. The constructed master chronology based on chemical and physical laminae falls entirely within the U–Th uncertainties as shown in Fig. 5, and confines the uncertainty bounds of U–Th. These uncertainties are large due to low U concentration and the elevated and variable initial ^230^Th/^232^Th activity ratio and thus the lamina-based chronology had to be used to improve dating accuracy. The average uncertainty of U–Th ages included in the age model is ca. 50%, whereas the initial lamina chronology has a maximum error of 15 years (4%), thus decreasing the uncertainty by at least 45%. By tying the U–Th ages to the annual lamina chronology a discrepancy was found, which was used to reconstruct the initial ^230^Th/^232^Th for each sample analysed, which ranged from 4.9 to 8.1 (median of 6.4, standard deviation of 0.9). The differences in initial ^230^Th/^232^Th are inferred to reflect variable mixing between a crustal Th component close to 0.8 derived from the basalt core of the island and a radiogenic reef limestone component which could be as high as 20 (e.g.^8^).

### Application of lamina chronology to reconstruct infiltration time series for Pu17

**Figure 6:**
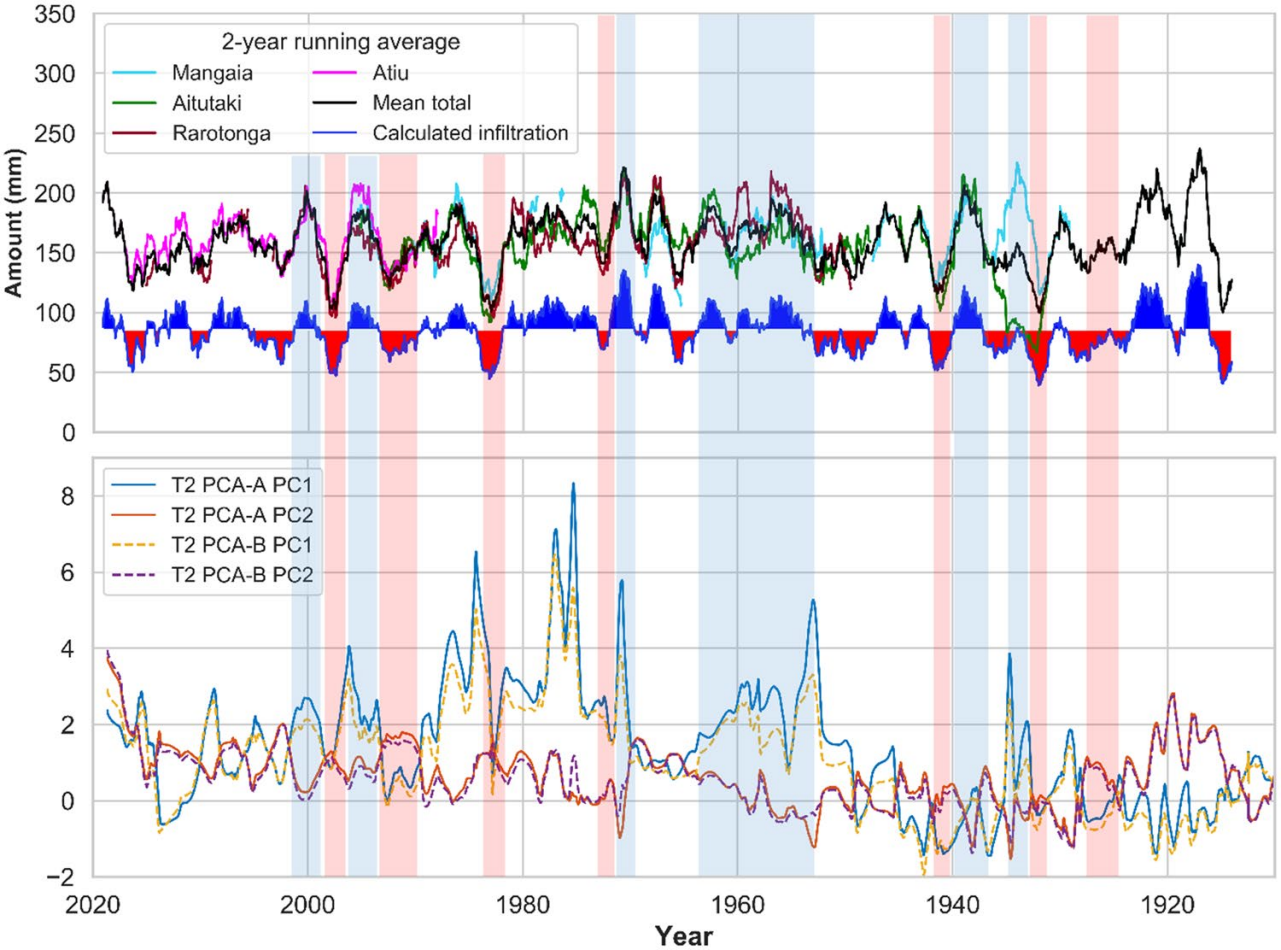
Comparison of principal components of trace elements with local infiltration record (1914–2019). Local infiltration was calculated by subtracting the potential evapotranspiration (PET) from the rainfall amounts at each island. The infiltration values of Mangaia, Aitutaki and Rarotonga were averaged and then tied with Atiu’s record. The blue (red) shadings of the calculated infiltration indicate positive (negative) anomalies from mean values of the period 1914–2019. The shaded light blue and light red vertical bands highlight the relatively wet and dry periods, respectively that are reflected in the principal components. PC1 of PCA-A correlates positively with infiltration values, whereas PC2 correlates negatively.

The adjusted master chronology and the conversion of speleothem depth-from-surface values to time (staring from March 2019, see Fig. 5) provided the basis for an accurately dated time series of trace element variability. The time series of trace elements and principal components were compared to local infiltration record, which suggests covariance between the two series for the whole 1914–2019 interval (Fig. 6). The shaded blue and red areas in Fig. 6 highlight relatively wet and dry periods, respectively. There is a negative correlation between PCA-A PC2 (aridity axis) and infiltration. Relatively dry and wet periods are characterised by higher and lower values of PC2, respectively. These emphasize the hydrological importance of trace elements in Pu17. The PC2 of PCA-A includes mostly bedrock/marine derived trace elements and has been interpreted as relatively low drip rate. The negative correlation between this PC and infiltration corroborates this interpretation. On the other hand, a positive correlation between PC1 of PCA-A (which includes Y and P) and infiltration supports the association of P and Y with relatively higher infiltration events.

**Figure 7:**
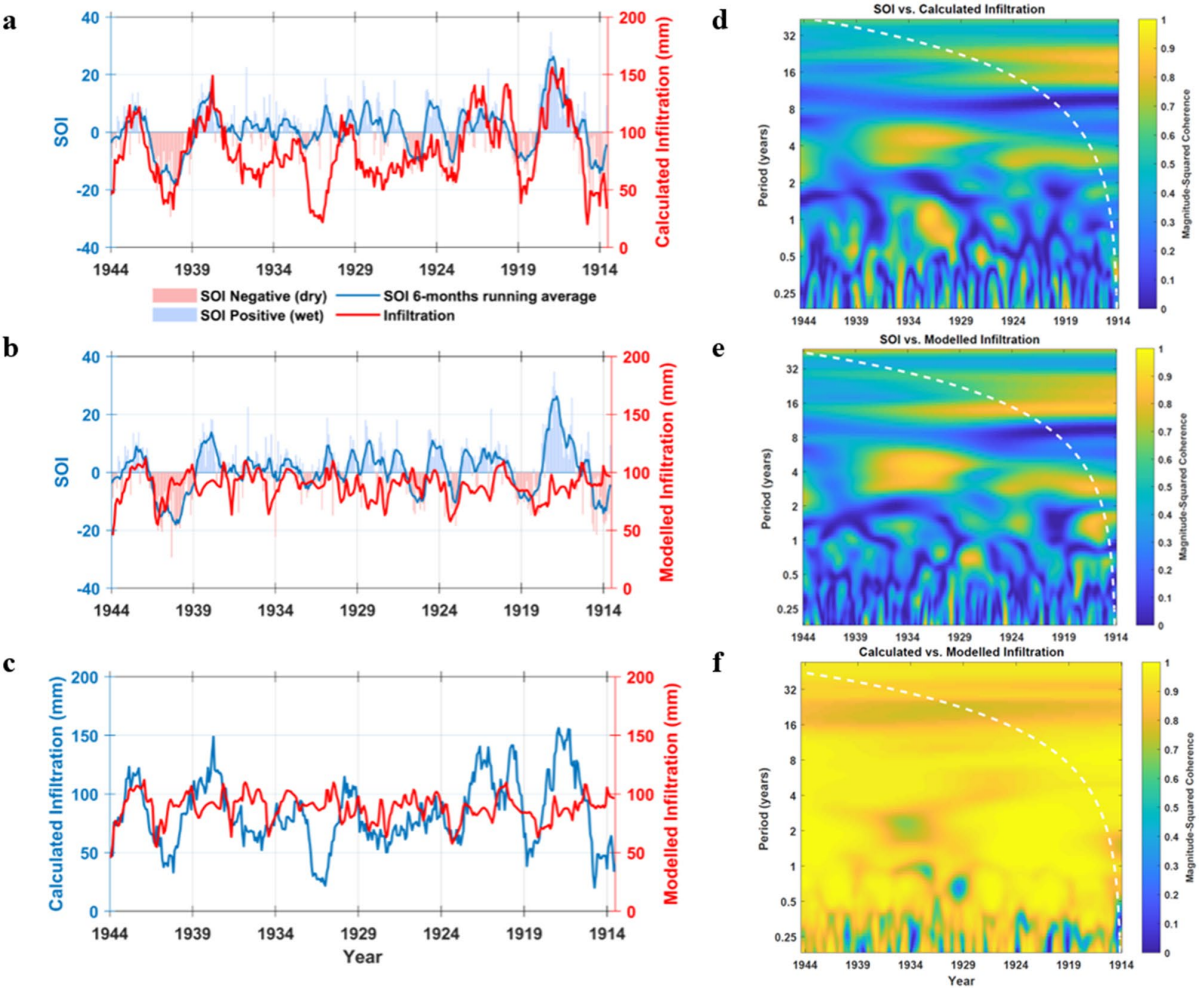
(**a**–**c**) Comparison of the records of SOI, calculated, and modelled infiltration for the time interval of 1914–1944. This interval was excluded from the GPR model and was used as test data to validate the model. As shown, the modelled infiltration follows the general trend of calculated infiltration and SOI. Over the period where calculated infiltration shows some discrepancy with SOI, modelled data match noticeably with SOI. (**d**) The coherency/correlation between SOI and calculated infiltration, (**e**) the coherency/correlation between SOI and modelled infiltration. The calculated and modelled infiltration show similar correlation with SOI for the periods of 2–8 years. (**f**) The wavelet coherency between calculated and modelled infiltration that highlight coherency values greater than 0.7 for the whole interval and at all periods. Dashed white curve on the coherency maps shows the wavelet’s cone of influence.

The next step is the use of Gaussian Process Regression (GPR) to explore the relationship between infiltration and trace elements time series. Our model provides a regression on the dataset with R^2^ equal to 0.83 and root mean squared error (RMSE) of 10.07. The mean squared and absolute errors (MSE and MAE) are 101.5 and 7.1, respectively. A continuous record of infiltration for the 347 years of Pu17 growth was then obtained. The records of SOI, the calculated and the modelled infiltration for the time interval of 1914–1944 were compared to each other in Fig. 7a–c. A correlation between these time series is expected because rainfall, infiltration into cave and drip rate, are all closely related to the SOI in the Cook Islands, due to the link between sea level pressure and local convection and precipitation^60^. It is apparent that the calculated infiltration follows the general trend of SOI for most of the record (Fig. 7a), however, there are intervals (1924–1934) where infiltration does not reflect exactly the variations in SOI. Such discrepancy could be due to how the infiltration was calculated. Figure 7b shows the comparison between SOI and modelled infiltration. It is evident that prominent wet and dry periods in the 6-month moving average of SOI are readily discernible in the modelled infiltration. The modelled infiltration matches SOI well over the 1924–1934 interval, for which calculated infiltration show some discrepancy. Modelled and calculated infiltration (Fig. 7c) do not completely match and the GPR model does not perfectly estimate the absolute values of infiltration. This could be due to the fact that the GPR model was applied without corrections for the marine aerosol contribution of Mg, and this likely resulted in the mismatch between absolute values for calculated and modelled infiltration. The exact quantification of the infiltration then requires an approach that takes into account different sources of trace element provenance and speleothem incorporation mechanisms. Given that marine aerosols contribute significantly to the concentration of trace elements in Pu17, wind (direction and speed) is another factor that would affect the incorporation of Mg. Wind provenance and speed is not directly connected to the amount of infiltration and this can be one of the reasons of the discrepancy between the calculated and the modelled infiltration values. Therefore, an accurate quantification of infiltration based on trace elements alone is not feasible for Pu17, and other independent proxies, such as oxygen isotope ratios and monitored crystal growth data should be accounted for. However, the aim of the present study is to use the modelled infiltration for advancing accuracy of an age model based on chemical/physical laminae that have been robustly correlated to annual hydroclimate cycles. The annual structure of infiltration is well reproduced by our reconstruction and the estimated values can be confidently used for the purposes of this study. The coherency between SOI and calculated infiltration is similar to the one between SOI and modelled infiltration for the periods of 2–8 years as highlighted by wavelet coherence analysis (Fig. 7d,e). In the interval from 1929 to 1934 there is a strong correlation (magnitude-squared coherency greater than 0.9) between the SOI and calculated infiltration for a 3–5 years period, and similarly between SOI and modelled infiltration. Despite a small difference between coherency maps, the modelled infiltration has captured coherent periodicity around 2–8 years and has replicated the periodicities observed in the calculated infiltration (Fig. 7f). Thus, wavelet analysis provides robustness to our inference that physical and chemical cycles are annual and their counting provides a robust chronology.

## Conclusions

We developed the first accurate age model for a low seasonal contrast, tropical South Pacific stalagmite affected by elevated and variable initial [^230^Th/^232^Th] ratio based on a two-dimensional, high-resolution physical and chemical mapping of laminae. This was complemented by parallel, multiple trace elements line scan PCs, which allowed selection of the clearest and most consistent annual signal that is necessary to build a master chronology and exclude proxy time-series that exhibit erratic behaviour.

Hydrologically-significant elements were found to be grouped in the “relatively dry” Mg, Sr, Na, Ba and the “relatively wet” Y and P. Their PCs were also used as a proxy, because they assign an unequivocal seasonal significance to each element within the annual hydrological cycle. It was thus possible to improve the chronology constructed on physical and chemical laminae by synchronising the laminae dataset to the local climate for the instrumental period record. The final synchronisation required a minimal adjustment (11 years over 336 years = 3%), thereby demonstrating the suitability of our approach to improve speleothem dating when there are uncertainties in the U–Th dating method.

Synchrotron-radiation based 2D maps of Sr concentration distribution, which is confirmed to be an excellent marker of relatively low drip rate (infiltration), tied to its relation to fabrics proved to be fundamental where laminations are less prominent than in Mediterranean and monsoonal settings characterized by high seasonal contrast. Physical and chemical mapping of laminae also allowed us to evaluate errors commonly encountered when lamina counting is based on LA-ICP-MS one-dimensional scans in terms of probability via assessing the prominence and lateral continuity of growth laminae.

Here, we advanced the empirical lamina counting method, into a technique that can be generally applied even for “low seasonal contrast” settings. We here demonstrated that the annual nature of chemical and physical laminae can be tested through their hydroclimate significance by comparing trace-element based chronology with calculated local infiltration data and one of its drivers, i.e., the SOI.

Our study shows that annual dating is possible even for stalagmites that formed under minimal hydrological contrast throughout the year opening up the possibility to obtain ages for many tropical speleothems affected by large dating uncertainties.

It stands to reason that a deeper understanding of how annual physical/chemical variability is generated in stalagmites from low-seasonal contrast settings (non-monsoonal tropics) may become a powerful method of dating which ultimately advances the potential of speleothems as unparalleled climate archives.

## Methods

### Monitoring

Given the remoteness of the Cook Islands and complex subdivisions of the land amongst custodians who do not dwell any more on the islands, a long-term on-site monitoring was not possible. In addition, there was no trained local individual who could operate an on-site monitoring station. These factors led to lack of a continuous and long-term on-site cave monitoring for Pouatea. However, cave temperature and relative humidity were measured hourly by using TinyTag TGP-4500 data loggers which were left in the cave. Cave air pCO_2_ was measured during two visits using a Vaisala MI70 Measurement Indicator equipped with GMP222 probe, with measurement accuracy of ± 3.5%. Drip rates at two drip points in another passage close by, parallel and well-interconnected with the passage where Pu17 was collected were measured hourly by using TinyTag TGP-4500 data loggers (2017–2018). Drip rate for Pu17 was measured manually by using a stopwatch during the course of two field trips in March and September 2019. Calcite growth experiments were conducted in-situ on glass slides for two years and via “instant” growth experiments for 30 min to 8 h on 3-mm in diameter Transmission Electron Microscopy grids (carbon coated) during “wet” and “dry” seasons.

### Trace element analysis

Trace element analyses were carried out at the School of Earth Sciences, University of Melbourne, using a 193 nm ArF excimer laser-ablation system coupled to an Agilent 7700 quadrupole ICPMS^61^. Element concentrations were measured from pre-ablated surface using a rectangular (200 µm × 20 µm) laser spot shape scanned parallel to the speleothem growth axis at a speed of 20 µm/s and a laser pulse rate of 10 Hz generating data-points every ∼ 6 μm. Analyses were conducted on five parallel scans repeated at a lateral offsets of 5 mm. The repeated tracks allowed identification of outliers caused by photomechanic ablation artifacts, which were then removed from the data. Quantification was carried out using NIST610 (Mg) and NIST612 (all other elements) glass as external standard. Raw spectrometry data were then reduced using Iolite software and internally normalised to ^43^Ca.

Synchrotron radiation micro-X-ray fluorescence (SR-µXRF**)** microscopy was performed at the XFM beamline at the Australian Synchrotron^62^ equipped with a Maia 384 detector array mounted 10 mm away from the sample target. The beam spot size was 1.5 μm and the monochromatic incident energy was set at 18.5 keV. After the acquisition of a 8 mm wide medium resolution (20 μm resolution) map, a 2.4 mm wide and 50 mm long 5 μm resolution map (481 × 10,002 pixels image) was obtained parallel to the stalagmite growth direction. The dwell time of the beam on each pixel was 10.9 ms allowing the detection of Ca, Fe, Br and Sr, with attenuation depths of 6 µm (Ca), 27 µm (Fe), 150 µm (Br) and 240 µm (Sr). The XFM spectral data were analysed using the GeoPIXE software suite, quantified by using single element Mn, Fe and Pt foils (Micromatter, Canada) and corrected by using a Ca matrix factor^63^.

### Radiometric dating

U–Th dating was carried out on 10 powdered samples (~ 200 mg) drilled from the stalagmite growth axis. The samples were dissolved in nitric acid, spiked using a ^229Th^–^233^U–^236^U tracer and U and Th were eluted in selective ion exchange resin following the procedure in Hellstrom^7^ and Drysdale et al.^64^.The measurements were performed using a Nu Instruments Plasma MC-ICP-MS at the University of Melbourne (Supplementary information, Table S-1). The U content varies between 20 and 100 ppb as is the case of active and modern stalagmites from Atiu. A most likely initial ^230^Th/^232^Th activity ratio of 6.12 ± 0.84 was determined after the exclusion of two outliers using the stratigraphic constraint procedure of Hellstrom^49^ and an age-depth model was determined from the remaining eight analyses using the finite positive growth rate model of Hendy et al.^50^ and the assumption of active growth at time of collection.

### Microstratigraphy

Stalagmite Pu17 was embedded in epoxy in order to avoid damage to the growing surface, cut along its vertical growth axis. The polished halves were imaged by using an Epson Expression 11000XL scanner at resolution of 2540 dpi, which highlights fabric changes across the scale of the whole specimen. Rapid recognition of fabrics was conducted by standard optical microscopy in plane polarized light and cross polarized light observations of 30 µm thick petrographic thin sections.

## Supplementary Information

Supplementary Information
