# Supplementary material for: Accurate dating of stalagmites from low seasonal contrast tropical Pacific climate using Sr 2D maps, fabrics and annual hydrological cycles
Source: Sci Rep. 2021 Jan 26;11:2178. doi: 10.1038/s41598-021-81941-x (PMC7838293; doi:10.1038/s41598-021-81941-x)
Supplement: Supplementary file 1 — Supplementary Information [file 41598_2021_81941_MOESM1_ESM.pdf]

Supplementary information:

Accurate dating of stalagmites from low seasonal contrast tropical Pacific climate using Sr 2D maps, fabrics and annual hydrological cycles

Mohammadali Faraji[1*], Andrea Borsato[1], Silvia Frisia[1], John C. Hellstrom[2], Andrew Lorrey[3], Adam Hartland[4], Alan Greig[2], David P. Mattey[5]

1. School of Environmental and Life Sciences, The University of Newcastle, NSW 2308, Australia
2. School of Earth Sciences, The University of Melbourne, VIC 3010, Australia
3. National Institute of Water and Atmospheric Research Ltd., Auckland 1149, New Zealand
4. Environmental Research Institute, School of Science, Faculty of Science and Engineering, University of Waikato, Hamilton 3240, New Zealand
5. Department of Earth Sciences, Royal Holloway University of London, Egham, Surrey, TW20 0EX, UK

*Correspondence to: M.F. (mohammadali.faraji@uon.edu.au)

Table S- 1, *results of multi-collector ICP mass spectrometry U-Th analyses.*

Sample	Lab Number	Depth(mm)	U(ngg^{-1})	[^{230}Th/^{238}U]^{a}	[^{234}U/^{238}U]^{a}	[^{232}Th/^{238}U]	[^{230}Th/^{232}Th]	Age(ka)^{b}	[^{234}U/^{238}U]$_{i}^{c}$	[^{230}Th/^{232}Th]$_{i}^{d}$
*Pu17-1	UME190524-207	6.0(2.0)	90	0.01740(35)	1.0263(20)	0.002117(42)	8.2	0.48(0.20)	1.0263(20)	8.05(0.24)
Pu17-2	UME190524-215	11.2(2.0)	118	0.00617(19)	1.0223(19)	0.000912(18)	6.8	0.067(0.086)	1.0223(19)	6.00(0.25)
Pu17-3	UME190524-244	15.2(2.0)	133	0.00598(16)	1.0221(18)	0.000837(17)	7.2	0.093(0.079)	1.0221(18)	5.98(0.27)
Pu17-4	UME190524-248	19.7(2.0)	104	0.00758(24)	1.0334(20)	0.000954(19)	7.9	0.187(0.089)	1.0334(20)	6.63(0.25)
*Pu17-5	UME190524-249	29.4(2.0)	81	0.06950(80)	1.0166(21)	0.00875(17)	7.9	1.82(0.84)	1.0167(21)	7.74(0.18)
Pu17-6	UME190524-291	34.5(2.0)	110	0.01310(27)	1.0212(19)	0.001665(33)	7.9	0.31(0.16)	1.0212(19)	6.55(0.21)
PU17-10	UME191209-420	38.4(2.0)	108	0.00477(21)	1.0224(18)	0.000387(08)	12	0.260(0.041)	1.0224(18)	6.01(0.50)
Pu17-7	UME190524-292	39.3(2.0)	115	0.00528(18)	1.0280(19)	0.000444(09)	12	0.275(0.045)	1.0280(19)	6.21(0.46)
Pu17-8	UME190524-296	45.0(2.0)	113	0.00859(24)	1.0329(19)	0.000828(17)	10	0.375(0.077)	1.0329(19)	6.87(0.27)
PU17-9	UME191209-416	48.2(2.0)	104	0.00409(19)	1.0283(18)	0.000204(04)	20	0.303(0.028)	1.0283(18)	4.88(0.97)

[a] Activity ratios determined at the University of Melbourne after after Hellstrom [1] and Drysdale, et al. [2].
[b] Age in kyr before year of measurement (2019), corrected for initial ^{230}Th using eqn. 1 of Hellstrom [3], assumed initial [^{230}Th/^{232}Th] of 6.12 ± 0.84 and the decay constants of Cheng, et al. [4].
[c] Initial [^{234}U/^{238}U] calculated using corrected age.
[d] initial [^{230}Th/^{232}Th] required to bring U-Th age into agreement with lamina counting agefor same depth.
2-s uncertainties in brackets are of the last two significant figures presented.
*outliers.

Table S- 2, *details of principal component analysis in each transect.*

PCA	Principal Component	Details	Transect	Mg	Sr	Ba	Na	Y	P	Variance
**PCA-A**	PC1	Including six elements (Mg, Sr, Ba, Na, Y, P)	**T1**	-0.21	0.46	0.54	-0.30	0.23	0.54	**40.38%**
			**T2**	-0.21	0.44	0.51	-0.29	0.37	0.51	**43.50%**
			**T3**	-0.18	0.44	0.52	-0.24	0.35	0.5	**42.16%**
**PCA-A**	PC2	Including six elements (Mg, Sr, Ba, Na, Y, P)	**T1**	0.70	0.28	0.32	0.55	-0.02	0.03	**23.66%**
			**T2**	0.65	0.25	0.35	0.54	-0.22	0.16	**24.30%**
			**T3**	0.63	0.23	0.36	0.54	-0.30	0.11	**26.11%**
**PCA-A**	PC3	Including six elements (Mg, Sr, Ba, Na, Y, P)	**T1**	-0.15	-0.30	0.17	0.30	0.85	-0.16	**14.37%**
			**T2**	0.10	-0.29	0.11	0.35	0.84	-0.22	**12.55%**
			**T3**	-0.20	-0.49	0.09	0.63	0.51	0.19	**11.91%**
**PCA-B**	PC1	Including four elements (Mg, Sr, Ba, Na)	**T1**	-0.28	0.61	0.58	-0.43	-	-	**42.12%**
			**T2**	-0.28	0.61	0.58	-0.44	-	-	**42.92%**
			**T3**	-0.06	0.67	0.68	-0.26	-	-	**41.08%**
**PCA-B**	PC2	Including four elements (Mg, Sr, Ba, Na)	**T1**	0.68	0.33	0.37	0.52	-	-	**34.59%**
			**T2**	0.66	0.30	0.41	0.53	-	-	**35.13%**
			**T3**	0.71	0.09	0.22	0.65	-	-	**36.72%**

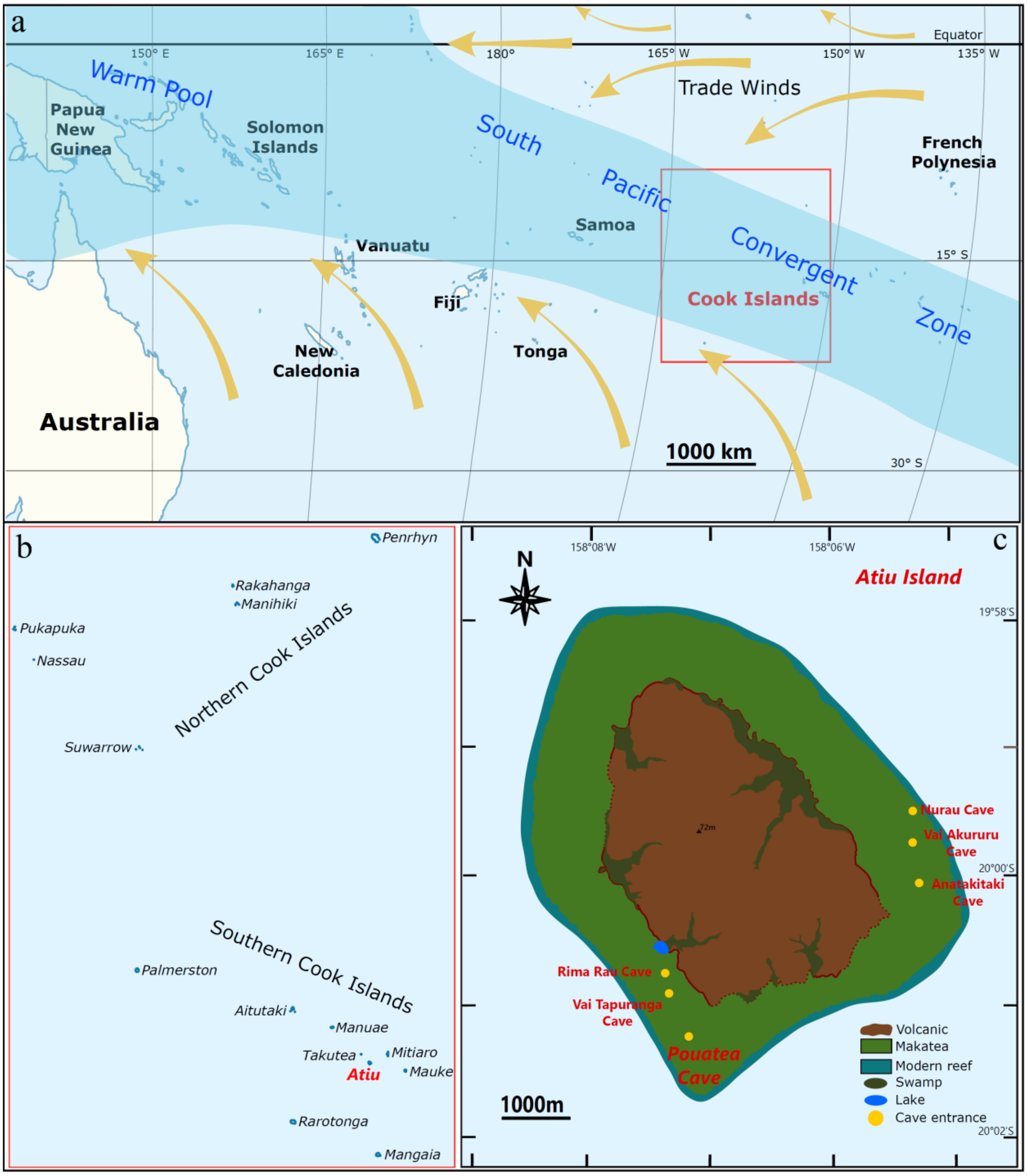

Fig S- 1: *a, SPCZ, and trade winds in the South Pacific Ocean (redrawn after Australian Bureau of Meteorology and CSIRO* [5]*). b, the Northern and Southern groups of the Cook Islands (red inset in a, redrawn after Australian Bureau of Meteorology and CSIRO* [6]*). c, Geomorphological map of Atiu Island (redrawn after Stoddart, et al.* [7]*) with the location of main caves. All maps were drawn using free software INKSCAPE v0.92 available at https://www.inkscape.org.*

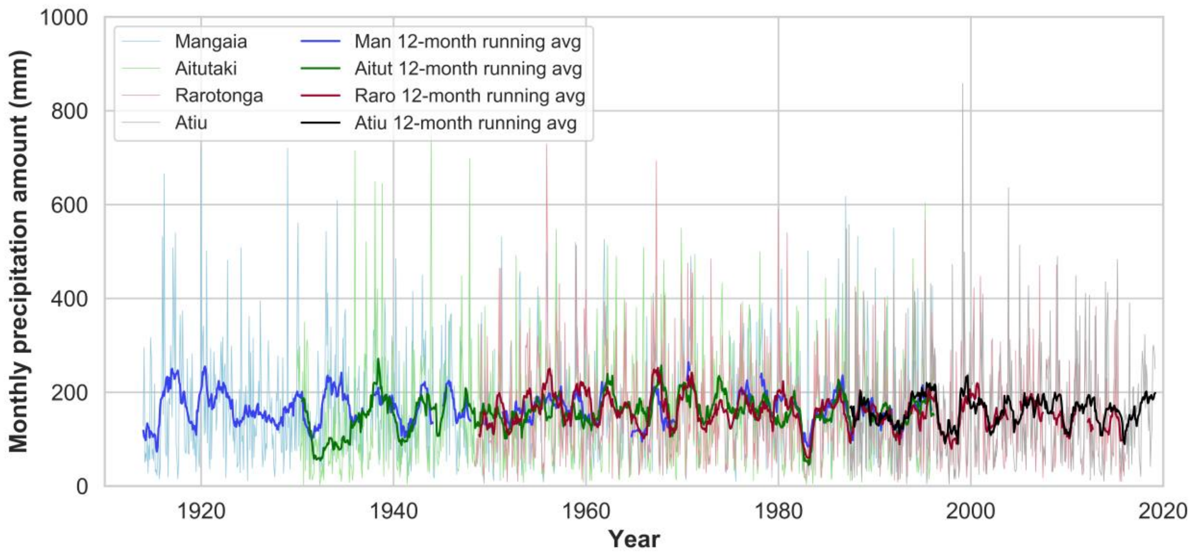

Fig S- 2: *local records of monthly precipitation and corresponding 1-year running average for four SCI stations: Mangaia (Jan 1914-May 1996), Aitutaki (Jan 1930-Jun 1996), Rarotonga (Jun 1948-Feb 2016) and Atiu (Oct 1980-Mar 2019). Despite differences in onset of meteorological data records, there is a consistency in the precipitation data patterns where records overlap.*

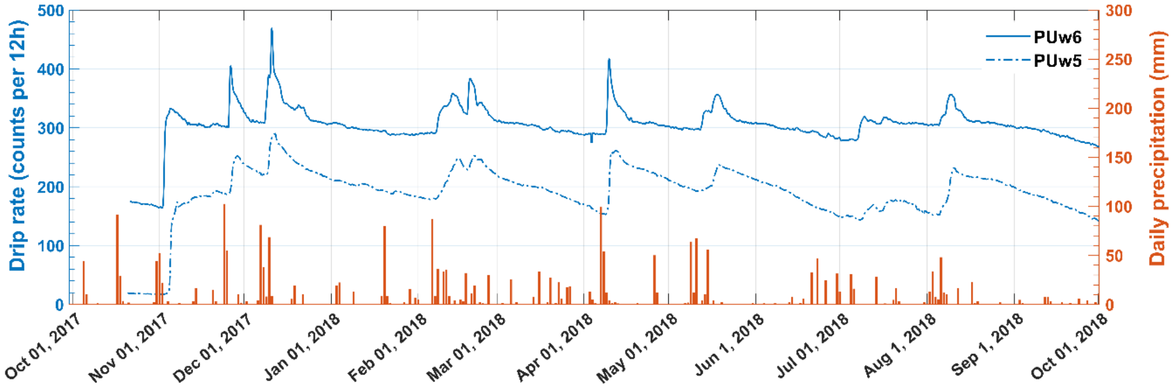

Fig S- 3: *drip rates for two drip points in Pouatea southern gallery compared with daily precipitation amount in Atiu. Drip rate variability responds to precipitation events within few days suggesting a short residence time in the aquifer.*

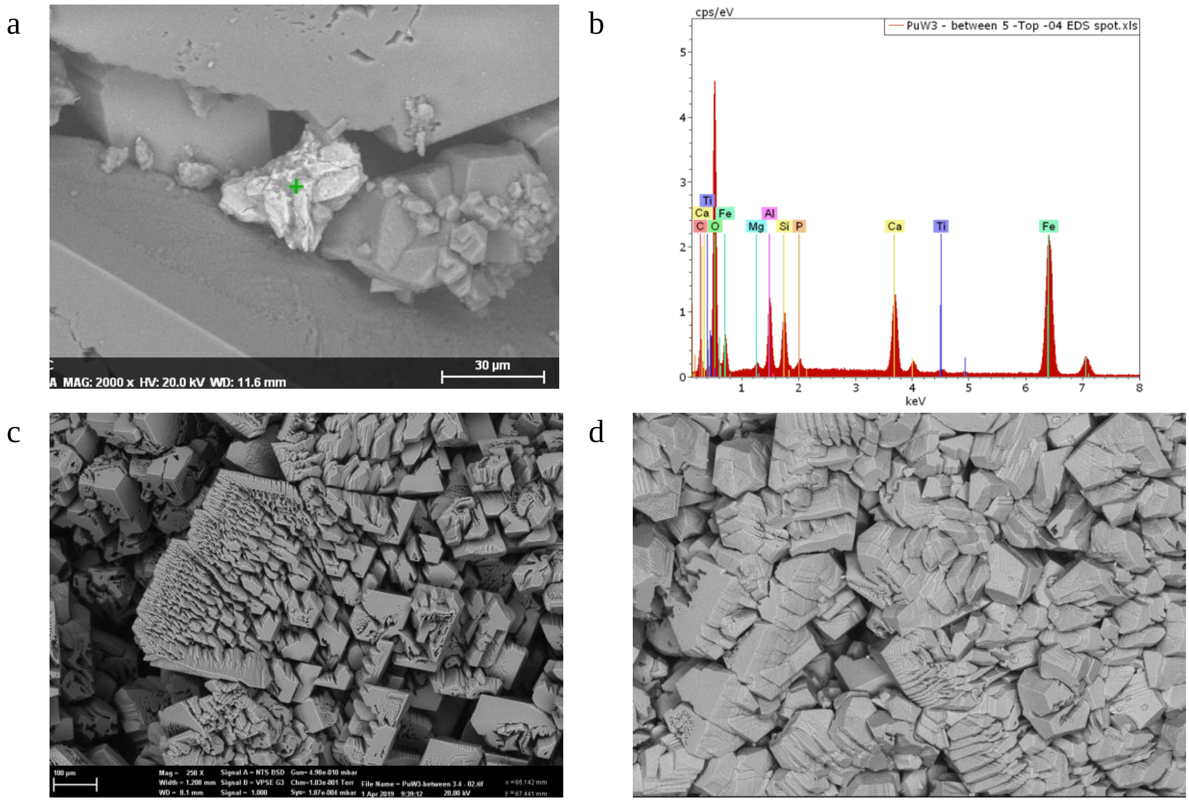

Fig S- 4: *a, particulate trapped between calcite rhombohedra (lighter under Back Scattered Electron mode) and b, its Energy Dispersive X-ray spectrum showing that trace elements such as P and Mg may be associated with typical inorganic colloidal particulate (Fe, Al, Si, Ti). Particulate is more commonly nested between growing crystals during enhanced infiltration. c, is the typical aspect of more open (porous) fabric, showing that porosity characterizes single rhombohedra, which are likely composite crystals. The frayed external rim of the central crystal was likely produced by transition from completely micro-phreatic conditions to more evaporative conditions; d, morphology of the more compact fabric (same scale as c) showing well developed rhombohedra, with lower intracrystalline porosity. Intercrystalline porosity is also being filled, so that boundaries between crystals impinge.*

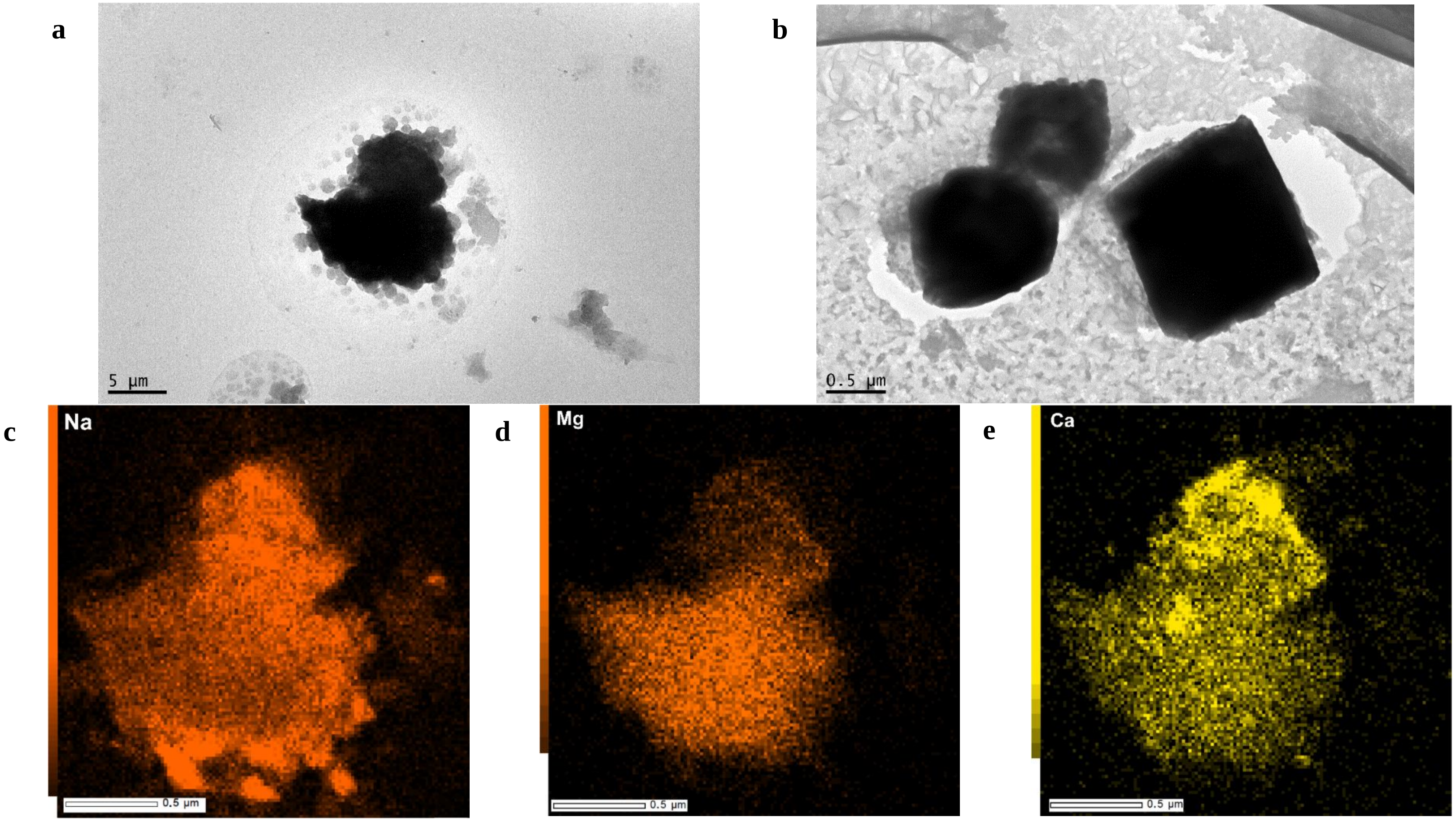

Fig S- 5: Results of in-situ precipitation experiments from Pouatea dripwater. *The experiment protocol follows Frisia, et al.* [8]*. Freshly collected dripwater was dripped onto a 3 mm carbon coated Transmission Electrom Microscopy (TEM) grid held at the tip of an inverted, sterilized stainless steel tweezer. The grid (still at the tip of the tweezer) was then inserted in a 0.2 mm centrifuge vial to avoid as best as possible evaporative effects and allow to degas for 4 hours to mimick precipitation from slow drip rates. The retrieved grid was then rinsed with deionized water and store in a cooler until ready for investigation. Both particulate (a) and NaCl cubic crystals (b) precipitated on the carbon coating. EDS mapping of the particle shown in S-5a identified Na, Mg and Ca, with Mg associated with Ca but not with Na. This experiment shows that marine aerosol contribute Na, Cl and Mg loads to the dripwater and that their effects on the chemistry of the precipitates is more conspicuous for slow drip rates.*

a

b

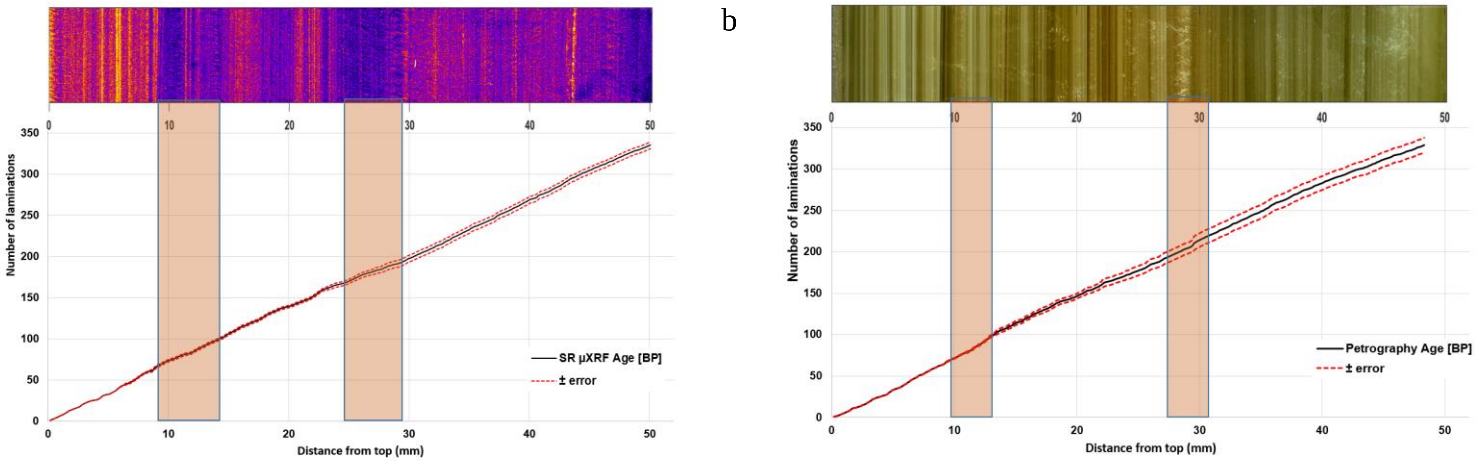

Fig S- 6, *laminae counting chronology and calculated ±errors for, a, the Sr synchrotron µXRF map,* ***b****, the high resolution scan of stalagmite. The shaded orange bars highlight the intervals where laminations are less clear, resulting in greater uncertainties in the age model. The ages are presented as Before Present, with the present being 2019.*

The records of Souther Oscillation Index (SOI) used in this study are accessable at:

1 – The Australian Bureau of Meteorology: http://www.bom.gov.au/climate/current/soi2.shtml

2 – Experimental Dendroclimatic Reconstruction of the Southern Oscillation [9].

References

1 Hellstrom, J. Rapid and accurate U/Th dating using parallel ion-counting multi-collector ICP-MS. *Journal of Analytical Atomic Spectrometry* **18**, 1346, doi:https://doi.org/10.1039/b308781f (2003).

2 Drysdale, R. N. *et al.* Precise microsampling of poorly laminated speleothems for U-series dating. *Quaternary Geochronology* **14**, 38-47, doi:https://doi.org/10.1016/j.quageo.2012.06.009 (2012).

3 Hellstrom, J. U–Th dating of speleothems with high initial 230Th using stratigraphical constraint. *Quaternary Geochronology* **1**, 289-295, doi:https://doi.org/10.1016/j.quageo.2007.01.004 (2006).

4 Cheng, H. *et al.* Improvements in 230Th dating, 230Th and 234U half-life values, and U–Th isotopic measurements by multi-collector inductively coupled plasma mass spectrometry. *Earth and Planetary Science Letters* **371-372**, 82-91, doi:https://doi.org/10.1016/j.epsl.2013.04.006 (2013).

5 Australian Bureau of Meteorology & CSIRO. Climate change in the pacific: Scientific assessment and new research (volume 1: Regional overview). (2011).

6 Australian Bureau of Meteorology & CSIRO. Climate Change in the Pacific: Scientific Assessment and New Research. Volume 2. Country Reports. (2011).

7 Stoddart, D. R., Woodroffe, C. & Spencer, T. Mauke, Mitiaro and Atiu: Geomorphology of makatea islands in the southern Cooks. doi:https://doi.org/10.5479/si.00775630.341.1 (1990).

8 Frisia, S., Borsato, A. & Hellstrom, J. High spatial resolution investigation of nucleation, growth and early diagenesis in speleothems as exemplar for sedimentary carbonates. *Earth-Science Reviews* **178**, 68-91, doi:https://doi.org/10.1016/j.earscirev.2018.01.014 (2018).

9 Stahle, D. W. *et al.* Experimental Dendroclimatic Reconstruction of the Southern Oscillation. *Bulletin of the American Meteorological Society* **79**, 2137-2152, doi:https://doi.org/10.1175/1520-0477(1998)079<2137:Edrots>2.0.Co;2 (1998).
